# Microbes on a Bottle: Substrate, Season and Geography Influence Community Composition of Microbes Colonizing Marine Plastic Debris

**DOI:** 10.1371/journal.pone.0159289

**Published:** 2016-08-03

**Authors:** Sonja Oberbeckmann, A. Mark Osborn, Melissa B. Duhaime

**Affiliations:** 1 Department of Biological Sciences, University of Hull, Cottingham Road, Hull HU6 7RX, United Kingdom; 2 School of Life Sciences, University of Lincoln, Brayford Pool Lincoln LN6 7TS, United Kingdom; 3 Environmental Microbiology Working Group, Leibniz Institute for Baltic Sea Research, Warnemünde, Germany; 4 School of Applied Sciences, Royal Melbourne Institute of Technology University, PO Box 77, Bundoora, VIC3083, Australia; 5 Department of Ecology and Evolutionary Biology, University of Michigan, Ann Arbor, Michigan, United States of America; University of Sydney, AUSTRALIA

## Abstract

Plastic debris pervades in our oceans and freshwater systems and the potential ecosystem-level impacts of this anthropogenic litter require urgent evaluation. Microbes readily colonize aquatic plastic debris and members of these biofilm communities are speculated to include pathogenic, toxic, invasive or plastic degrading-species. The influence of plastic-colonizing microorganisms on the fate of plastic debris is largely unknown, as is the role of plastic in selecting for unique microbial communities. This work aimed to characterize microbial biofilm communities colonizing single-use poly(ethylene terephthalate) (PET) drinking bottles, determine their plastic-specificity in contrast with seawater and glass-colonizing communities, and identify seasonal and geographical influences on the communities. A substrate recruitment experiment was established in which PET bottles were deployed for 5–6 weeks at three stations in the North Sea in three different seasons. The structure and composition of the PET-colonizing bacterial/archaeal and eukaryotic communities varied with season and station. Abundant PET-colonizing taxa belonged to the phylum Bacteroidetes (e.g. Flavobacteriaceae, Cryomorphaceae, Saprospiraceae—all known to degrade complex carbon substrates) and diatoms (e.g. Coscinodiscophytina, Bacillariophytina). The PET-colonizing microbial communities differed significantly from free-living communities, but from particle-associated (>3 μm) communities or those inhabiting glass substrates. These data suggest that microbial community assembly on plastics is driven by conventional marine biofilm processes, with the plastic surface serving as raft for attachment, rather than selecting for recruitment of plastic-specific microbial colonizers. A small proportion of taxa, notably, members of the Cryomorphaceae and Alcanivoraceae, were significantly discriminant of PET but not glass surfaces, conjuring the possibility that these groups may directly interact with the PET substrate. Future research is required to investigate microscale functional interactions at the plastic surface.

## Introduction

Plastic pollution was first reported in remote, offshore basins of the north Atlantic ocean over forty years ago [1, 2], twenty years after the introduction of plastic to the consumer market [3]. Research has shown that plastic debris is ubiquitous in aquatic habitats [4]. Estimates of total plastic load in the oceans are 5 trillion pieces of plastic weighing over 0.25 million tons [5], while estimates of plastic in surface waters are as high as half a million pieces per square kilometer [6]. Plastic pollution is established also in marine sediments [7] and in some of the planet’s largest [8–10] and most remote [11] reservoirs of freshwater. Based on a combination of data on solid waste management practices, population density and economic status, the input of plastic debris from land to the ocean is projected to increase by an order of magnitude over the next ten years [12].

As the spatial distribution of marine plastic debris continues to be better resolved, research is increasingly focused on assessing its effects on environmental and public health. The impacts of plastic pollution range from organismal, such as morbidity and mortality due to entanglement and intestinal blockage [13] to food web, as ingested plastic can pass to higher trophic levels [14] and can lead to energetic costs [15]. Further, there are toxicological effects, as microplastics dynamically adsorb and desorb hydrophobic organic pollutants [16–20] and carry additive-derived plasticizers [21]. Yet, one critical and underexplored knowledge gap is the role that rapidly colonizing plastic biofilm communities play in the impact of plastic debris on the marine ecosystem [22–24].

Plastic surfaces in seawater can form microbial biofilms visible by eye within one week and cause a physical change, with a significant increase in plastic hydrophilicity and a shift from positive towards neutral buoyancy after 2 weeks [25]. Yet, the ecosystem-level implications of these microbial colonizers are only speculated, with considerations ranging from microbes being pathogens [24,26–27], bloom-forming harmful algae [28], invasive species [29], or capable of degrading either the polymers or the adsorbed organic pollutants [24,26].

The first two studies to use high-throughput sequence analysis of 16S ribosomal RNA genes to describe microbial biofilm communities from open ocean [26] and urban river [27] plastics found plastic communities to be distinct from those in surrounding water. The authors of the North Atlantic study observed microbial cells in pits on the plastic surface, which led them to implicate plastic-associated microbes in potential degradation of the plastic surface [26]. A scanning electron microscope (SEM)-based study in Australian waters observed similar pits and grooves on the surfaces of marine plastic debris [30]. A recent microcosm experiment investigating initial biofilm formation on polyethylene in coastal sediments documented successional changes in bacterial community composition over a two-week period [31]. The study suggested selection for specific bacterial taxa, *Arcobacter* and *Colwellia*, which have previously been shown to affiliate with hydrocarbon contamination [31]. A substrate deployment study documented early successional changes in diatom communities on polyethylene and biodegradable plastic and signs of substrate-specific degradation of biodegradable plastic as early as two weeks [32]. In addition to substrate type, seasonal and spatial factors shape the microbial community associated with plastic surfaces in aquatic systems [33–35]. An exposure experiment that deployed a range of substrates in a river showed biofilm community structure to be influenced most strongly by deployment location [36], presumably due to differing environmental conditions. These studies have laid a foundation upon which to interrogate the factors driving the composition and structure of microbial communities on marine plastic debris.

The present study examines the influence of season, geographic location, seawater, and substrate material type on the microbial colonization of single-use plastic drinking bottles composed of poly(ethylene terephthalate) (PET) deployed at multiple stations in the North Sea. PET is a semicrystalline thermoplastic polyester that is commonly used in textiles and food and beverage packaging and comprises 50% of synthetic fiber production worldwide [37]. In this study we use high-throughput sequencing to describe bacterial, archaeal, and eukaryotic constituents of biofilms colonizing ocean plastic. We thereby build upon previous work that used low-throughput molecular fingerprinting to track course changes in the bacterial members of the biofilm communities [35]. We hypothesize that the plastic microbiome will be *(i)* distinct from free-living (0.22–3 μm) and particle-associated (>3 μm) microbial communities in seawater and *(ii)* will be distinct from those colonizing another non-plastic chemically inert hard substrate (glass). We further hypothesize that *(iii)* patterns in community structure will differ significantly depending on site and season. Knowledge of the community composition and temporal and spatial factors influencing plastic-dwelling microbes is central to understanding the impact of these biofilm communities on the fate (e.g., potential for biodegradation) and ecosystem impact (e.g., vectoring pathogens, toxic, or non-native microbes) of plastic pollution in marine environments.

## Materials and Methods

### Experiment design and sampling

**Fig 1:**
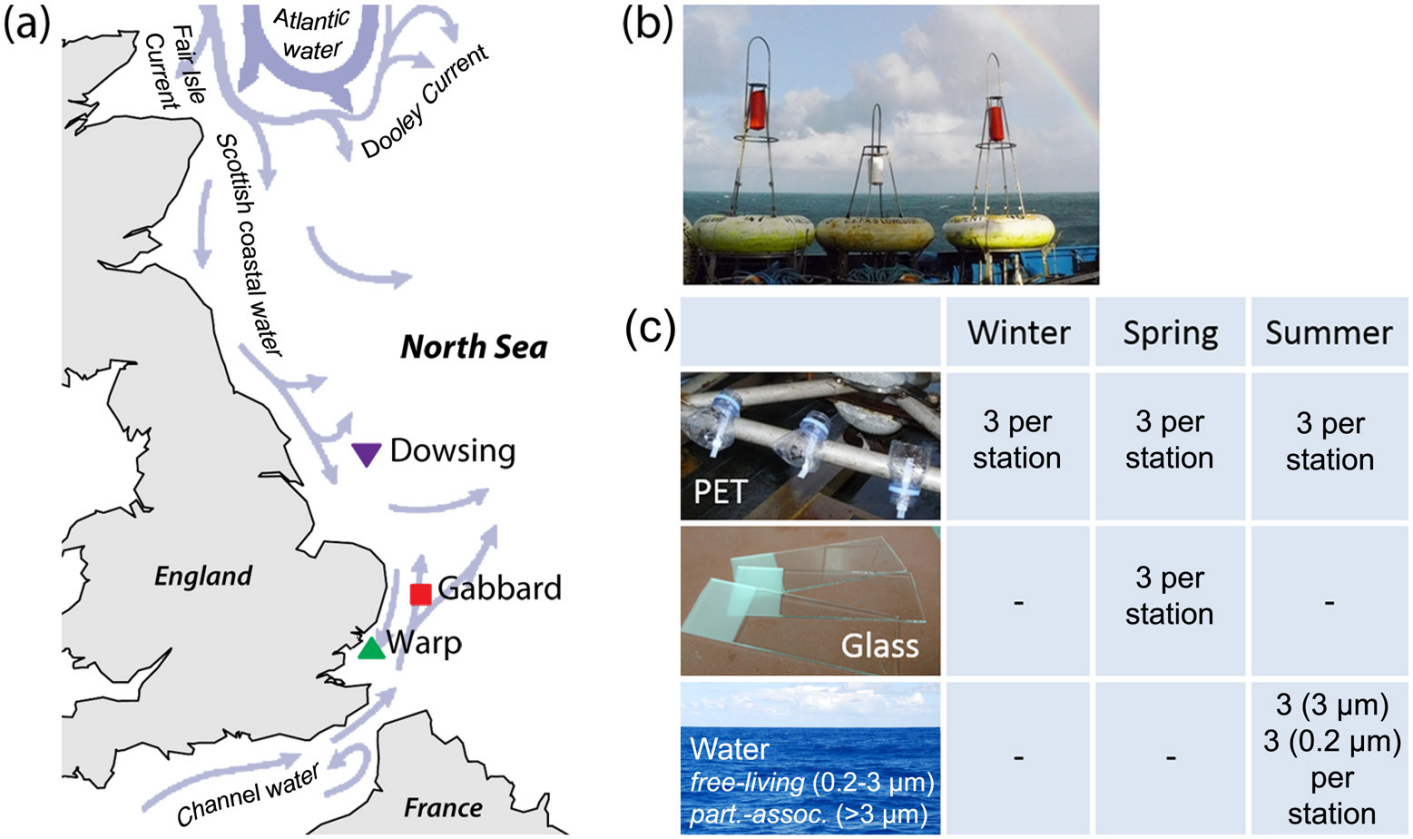
Experimental setup and map of North Sea (UK) including currents and sampling stations. (a) Map of SmartBuoy locations Dowsing (purple), Warp (green) and Gabbard (red), including dominant regional current systems (current flow information modified after [41]), (b) SmartBuoys from which PET bottles were deployed, including attachment setup, (c) samples and replicates included in this study.

An exposure experiment was designed to investigate spatial and seasonal dynamics of microbial biofilm communities attached to plastic fragments in marine waters. Six poly(ethylene terephthalate) (PET) drinking water bottles (Evian, 500 ml) were attached to three SmartBuoys [38] in the North Sea off the U.K. coast (Fig 1a and 1b) in three different seasons: winter (Dec 2011 to Jan 2012), spring (Mar to Apr 2012) and summer (Aug to Sep 2012). SmartBuoys are automated platforms that record marine environmental data hourly at fixed locations [38]. For this study, we collected temperature, salinity, and chlorophyll fluorescence data from the buoys over the course of the substrate recruitment experiment (S1 Table). The buoys employed were the Warp SmartBuoy (51°31.497N, 01°01.452E; ‘W’), the West Gabbard SmartBuoy (51°58.883N, 02°04.902E; ‘G’) and the Dowsing SmartBuoy (53°31.918N, 01°03.432E; ‘D’). Glass microscope slides were fixed as chemically inert hard non-plastic substrates at all three buoys in spring (Mar–Apr 2012) to determine the extent of plastic-specific microbial colonization and to identify PET-specific microorganisms. Due to strong currents and winds, some bottles were lost, but at least three biological replicates (i.e., three entire bottles) per season and station were recovered for molecular analysis (Fig 1c). To generate a map of the buoy deployments, sample site coordinates were mapped in R [39] using ‘maps’ package (v. 2.3–9; [40]). Current flows were manually overlaid in Photoshop CS3 (v 10.0.1, Adobe; CA, USA) based on [41].

Upon sample collection from the buoys, the PET samples were cut from the bottles using sterilized scissors and glass slides were retrieved whole. PET and glass were rinsed with sterile seawater. Plastic fragments of approximately 10 cm^2^ were cut from the bottles using sterilize scissors for DNA extraction. The plastic and glass samples were treated to remove ambient seawater, residual debris and non-attached organisms. The plastic samples were cut with sterilize scissors into pieces to fit in 25 ml tubes and centrifuged for 30 sec at 5,000 rpm. Glass samples were manually agitated in sterile seawater and briefly shaken off after washing. The plastic and glass samples were stored at -20°C until further analyses.

Seawater samples were collected at each station in summer (Sep 2012; due to sampling instrument access, water collection was possible in summer only). Niskin bottles attached to a sampling rosette were used to collect surface water (1 m) at each station. Collection bottles were rinsed three times with station water from the Niskins before holding water to be filtered. Water was then serially filtered in triplicate through 3 μm (1 liter) and 0.22 μm (500 ml) filters (47 mm, cellulose acetate). Filters were stored at -20°C until further analyses.

No privately owned or protected land requiring specific permits was accessed and no protected or endangered species were collected in this field sampling.

### Scanning Electron Microscopy

For qualitative assessment of biofilm structure, a random collection of 8 samples across all seasons and sites was chosen for scanning electron microscopy (SEM) and prepared for microscopy, modified after [42]. Plastic fragments were treated onboard with 2.5% glutaraldehyde in 0.1 M sodium cacodylate buffer (pH 7.4) for 2 h at 20°C and then washed two times for 30 min with 0.1 M sodium cacodylate buffer. The samples were dehydrated in a step-wise series of 15 min submersions in 75% ethanol (1x), 90% ethanol (1x), absolute ethanol (2x), and absolute acetone (1x) then stored in a negative pressure fume hood. The dehydrated plastic fragments were carbon coated with a SC7620 Mini Sputter Coater (Quorum Technologies, East Grinstead, UK) and images were taken with an Inspect S50 SEM (FEI, Hillsboro, OR, USA) at an accelerating voltage of 10.00–15.00 kV.

### DNA extraction

DNA was extracted from exposed PET bottles, glass slides, and filters. PET (0.5 g per sample) and filters (cut into several pieces) were transferred to a 2 ml tube containing TSE buffer (50 mM Tris, 6.7% sucrose, 1 mM EDTA; 700 μl total), incubated in lysozyme (0.3 mg/ml final; 37°C for 30–60 mins), then incubated at 50°C for 60 mins in Tris-EDTA (50 mM Tris, 250 mM EDTA, pH 8; 74 μl) and SDS buffer (20% [w/v] SDS, 20 mM EDTA, 50 mM Tris, pH 8; 44 μl). Tubes were centrifuged (8,000 g, 10 mins) to remove lysed biofilm and cellular material, the supernatant (approx. 920 μl) transferred to a new tube and 1/10 volume of NaCl and 1 volume phenol:chloroform added. Phenol:chloroform extraction and isopropanol precipitation were performed as described previously [43,44]. Glass slides were identically processed, but in 50 ml Falcon tubes adding 10.5 ml TSE buffer and adjusted volumes of the other reagents. DNA extraction yields were measured with a NanoDrop Spectrophotometer (Thermo Scientific; Wilmington, DE).

### Tag sequence amplification and Illumina MiSeq library generation

Dual-indexing was used to generate a barcoded MiSeq library of tag sequences (V4 region of 16S and V9 region of 18S rRNA genes, for bacteria and eukaryotes, respectively (see S2 Table for sequences of full constructs and [45] for details regarding their design). Gene-specific primers used to target the 16S V4 region were 515F (5’- GTGCCAGCMGCCGCGGTAA-3’; [46,47]) and 806R (5’-GGACTACHVGGGTWTCTAAT-3’; [47]) and for the 18S V9 region 1391F (5’-GTACACACCGCCCGTC-3’; [48]) and 1795R (*EukB*, 5’-TGATCCTTCTGCAGGTTCACCTAC-3’; [48,49]).

PCR amplification was carried out in 96 well plates, with each well containing 17 μl Accuprime Pfx Supermix (Life Technologies, NY, USA), 1 μL template DNA (20–100 ng; samples >100 ng were diluted 1:10, as the high yield inhibited PCR amplification) and 2 μl of each paired set of index primers [10 μM]. Sterile PCR grade water served as negative control and known DNA as positive control (16S: *Pseudoalteromonas* str. H105 [50]; 18S *Saccharomyces cerevisiae*). Thermal cycling (Mastercycler Nexus, Eppendorf; Germany) consisted of an initial denaturation of 95°C for 2 min, 30 cycles of 95°C for 20 sec, 55°C for 15 sec, and 72°C for 5 min, with a final extension at 72°C for 10 min. Successful amplification was confirmed for a random row of 12 samples per PCR plate by agarose gel electrophoresis (1% w/v in 1x TBE buffer at 100 V) and imaging of GelRed (Biotium, Inc.; CA)-stained DNA by UV light (302 nm; ChemiDoc MP, Bio-Rad Laboratories, Inc.; CA, USA). PCR cleanup and sample normalization to pool to equimolar concentrations were performed using the SequalPrep Normalization Plate Kit (Life Technologies, NY USA), per manufacturer guidelines. Library concentrations were quantified by qPCR using primers targeting the Illumina adapters (Library Quantification Kit, KAPA Biosystems, MA, USA) and mixed with non-indexed PhiX control libraries. Sequencing of paired-end reads was carried out on an Illumina MiSeq Personal Sequencer using a MiSeq Reagent Kit v2 for 500 cycles for ~250 bp reads (Illumina, CA, USA). All raw sequence files are available from the NCBI Short Read Archive (SRA) database (BioProject: PRJNA283545; BioSamples: SAMN03846994-SAMN03847011).

### Sequence data processing

Quality filtering of reads (permitted length = 225–275 bp for 16S, 100–180 bp for 18S sequences, maximum number of ambiguous bases per sequence = 0, maximum number of homopolymers per sequence = 8), taxonomy assignment (Bayesian classifier, reference database SSURef_119_SILVA, required bootstrap value ≥ 85%) and picking of operational taxonomic units, OTUs, (label = 0.03) were carried out using Mothur [51]. Chloroplasts, mitochondria, eukaryotes and unknown sequences were removed from the 16S analysis (“Chloroplast-Mitochondria-unknown-Eukaryota”), while bacteria, archaea, and unknowns were removed from the 18S analysis (“unknown-Bacteria-Archaea”). OTUs with a total abundance of 1 were excluded from downstream analyses. Gabbard PET summer samples were excluded from the analysis due to insufficient sequencing read coverage.

### Beta Diversity Analysis

For beta diversity analysis and related hypothesis testing, samples with less than the 5^th^ percentile of reads (<1110 reads for 16S, <800 reads for 18S) were removed from analyses, proportions of OTUs per sample were calculated (OTU count/total OTUs in sample), proportions were square root-transformed, and scaled by the minimum library size per subset. This work was performed in R [39] using the phyloseq package [52].

Permutational Multivariate Analysis of Variance, PERMANOVA [53], was used to test for significant differences between treatments, seasons, and stations based on a Bray-Curtis resemblance matrix and 999 permutations. Monte Carlo simulations were used to generate p-values when 999 unique permutations were not possible for all pair-wise tests. To give confidence that data in significant PERMANOVA results were not over-dispersed, homogeneity of dispersion (PERMDISP) was tested based on calculated distances to centroids [54]. Non-significant PERMDISP results supported the null hypothesis of equal within-group dispersions among groups. To visualize patterns of the different seasons, stations and treatments, principal coordinates analyses (PCO) were performed [55,56]. For PERMANOVA, PERMDISP and PCO, Primer 6 [57] with the PERMANOVA+ package (PRIMER-E Ltd, Plymouth, UK; [58]) was used.

Total community OTU representations across treatments were depicted in a taxonomic framework by importing OTU tables with their Silva [59] taxonomy string into MEGAN (v5; [60]) for visualization of relative abundance of top 25 most abundant taxa in the PET-colonizing communities and relative abundances of taxa in a phylogenetic tree framework.

### Significantly discriminant OTUs

To identify OTUs that discriminate the treatments, stations, and seasons, we used a linear discriminant analysis effect size method (LEfSe; [61]) on the software author-provided Galaxy framework (all default settings for data formatting and LDA effect size). LEfSe offers the ability to look for differentially abundant OTUs across ‘classes’ that are consistent across similar class types, or ‘subclasses.’ For instance, to identify discriminant OTUs between treatments that were consistent across all stations, treatments were set as LEfSe classes and stations set as subclasses. Class and subclass variables are noted in the results tables.

### PET community correlation analyses

For the metadata analyses, three environmental parameters collected from the SmartBuoys (temperature, salinity, chlorophyll fluorescence) were collated across each season for each station. The average values were calculated for each station-season combination; salinity data for Dowsing-Summer were not available. A Mantel test (R; mantel, vegan package [62]) was performed with 999 permutations to compare Euclidean distance matrices (R; dist) of environmental parameters (T, S, F) and Bray-Curtis dissimilarity matrices of the bacterial/archaeal 16S and eukaryotic 18S PET-colonizing OTU abundances (R; vegdist, vegan package). A second set of Bray-Curtis distance matrices of the 16S and 18S OTU tables of PET colonizing communities were generated based only on the samples shared by both 16S and 18S datasets. A Mantel test was performed with 999 permutations to compare these truncated 16S and 18S matrices (R; mantel).

## Results

### Plastic specificity of the PET Microbiome

**Fig 2:**
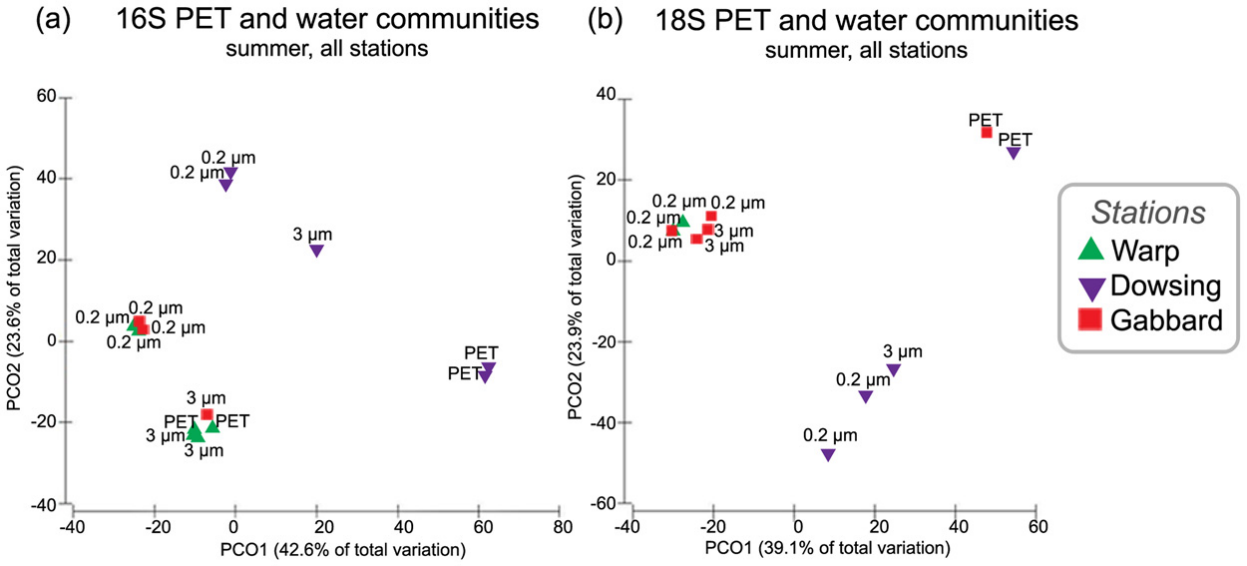
Principle Coordinate Ordinations relating variation in microbial community composition between plastic and seawater communities in summer. PCOs representing similarity of biofilm communities based on counts of OTUs across samples (16S/18S rRNA gene data, see methods for OTU definition). Displayed are comparisons of (a) bacterial/archaeal and (b) eukaryotic communities of PET-attached, particle-associated (>3 μm) and free-living (0.22–3 μm) seawater communities sampled in summer.

To determine whether microbes colonizing plastic are distinct from those in seawater and on other inert hard substrates, microbial community structure of PET biofilms was compared to free-living (0.22–3 μm) and particle-associated (>3 μm) communities in seawater and those colonizing glass. The 16S rRNA gene sequence comparisons showed a significant difference between the PET-colonizing and free-living bacterial/archaeal seawater communities (p = 0.009; pairwise PERMANOVA, S3 Table), but no significant difference between PET-colonizing and particle-associated communities (p = 0.377). The two size-fractionated seawater communities were significantly different from one another (p = 0.044). Within stations, samples clustered according to this free-living or particle-associated/“attached” dichotomy (>3 μm fraction seawater *or* PET-attached; Fig 2a). There was no significant difference between the glass and PET-attached communities (p = 0.058, Table 1).

**Table 1:** PERMANOVA/PERMDISP results of substrate, season and station-specific variation in PET microbial communities.

Subset: Factors	marker	df	SS	Pseudo F	p(perm)	Unique perm	p(MC)	p(PERMDISP)
PET: Station	16S	2	10987	2.5436	**0.001** *	999	**0.004** *	0.542
	18S	2	10258	2.8614	**0.001** *	999	**0.001** *	0.632
PET: Season	16S	2	14855	3.7968	**0.001** *	997	**0.001** *	0.281
	18S	2	9542.3	2.5881	**0.002** *	999	**0.004** *	0.794
Summer: Treatment (PET-3-0.2)	16S	2	9435.2	3.0775	**0.003** *	991	**0.011** *	0.125
	18S	2	12006	3.4363	**0.001** *	905	**0.007** *	0.956
Spring: Treatment (PET-glass)	16S	1	3746.9	1.9241	0.058	980	0.057	0.841
	18S	1	1968.7	1.1056	0.29	905	0.344	0.894
Summer: Station (Warp-Gabbard-Dowsing)	16S	2	10097	3.4277	**0.003** *	994	**0.008** *	**0.005** *
	18S	2	9426.7	2.2778	**0.026** *	937	**0.042** *	0.38
Spring: Station (Warp-Gabbard-Dowsing)	16S	2	9373.4	2.7822	**0.002** *	997	**0.001** *	0.848
	18S	2	12674	6.111	**0.001** *	997	**0.002** *	0.573
Summer: “Attached” versus Free-living	16S	1	6692.7	4.0966	**0.003** *	843	**0.013** *	0.124
	18S	1	5358.4	2.3385	**0.025** *	416	0.084	**0.023** *

PERMANOVA main tests compare both bacterial/archaeal and eukaryotic (16S and 18S rRNA gene, respectively, denoted by ‘marker’) community structure across seasons, stations, and treatments. Tests are displayed for three data subsets (PET, spring, summer). Significant results (p < 0.05) highlighted in bold and marked with *. P-values were obtained using type III sums of squares and 999 permutations [‘p(perm)’] or calculating Monte-Carlo tests [‘p(MC)’]. Pseudo F, PERMANOVA F statistic; d.f., degrees of freedom; SS, sums of squares; Unique perm, unique permutations. p(PERMDISP) are p-values of PERMDISP tests, calculated to centroids.

As with the bacterial and archaeal communities, the eukaryotic communities colonizing the PET significantly differed from the free-living seawater communities (pairwise PERMANOVA, p = 0.031, S3 Table; Fig 2b), but resembled the particle-associated communities (p = 0.092). There was no significant difference between the PET- and glass-colonizing communities for both bacterial/archaeal and eukaryotic communities (p = 0.058 and 0.29, respectively; Table 1).

### Distinctive members of the PET microbiome

**Fig 3:**
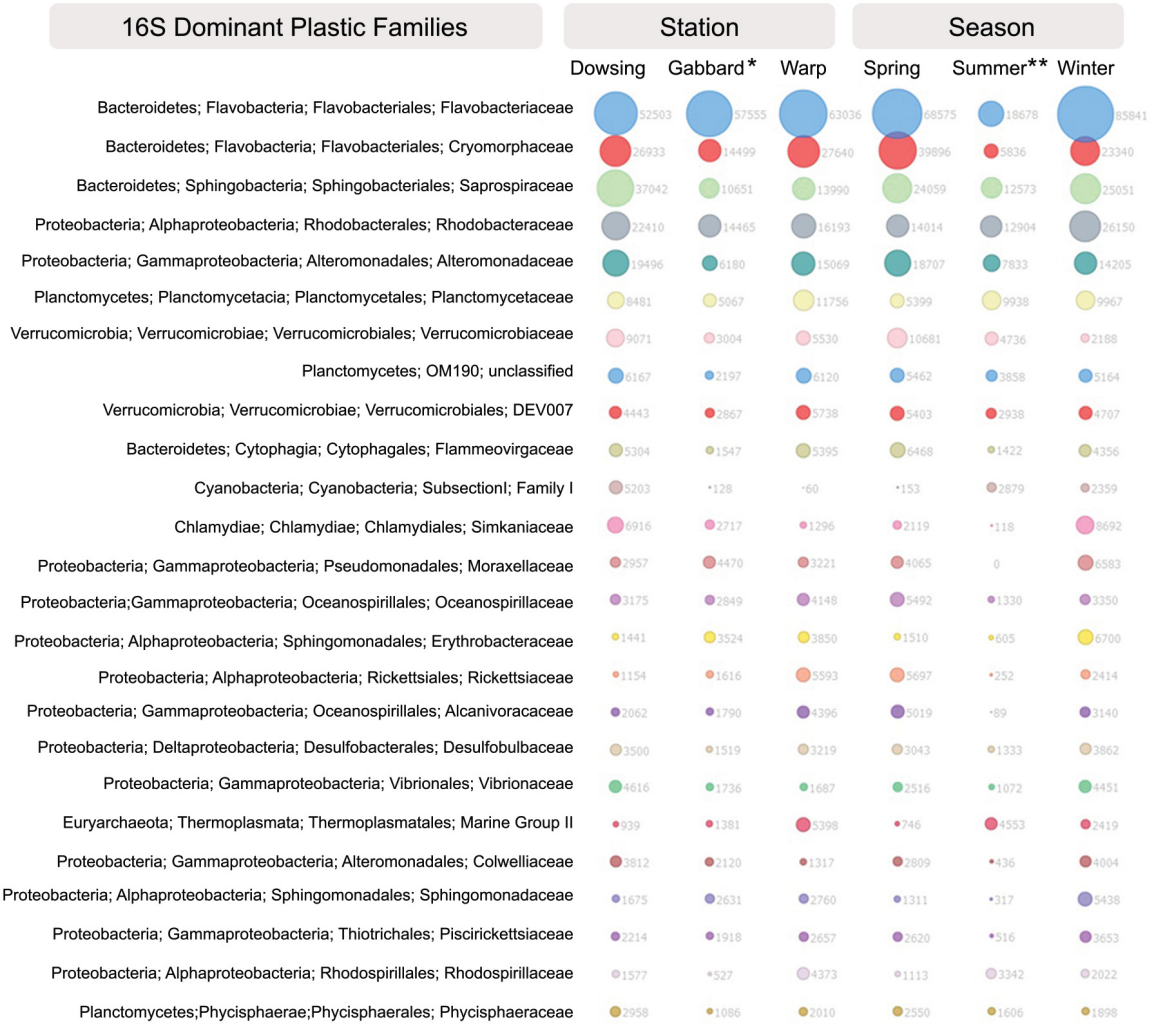
Abundant bacterial/archaeal families within PET communities. Most abundant (top 25) bacterial and archaeal families present in PET-attached biofilm communities after deployment in the North Sea, grouped per deployment site/station and season (based on 16S rRNA gene analysis). * Gabbard represents data for winter and spring only; ** summer represents Warp and Dowsing data only; Gabbard summer was removed from analysis due to insufficient sequencing effort.

**Fig 4:**
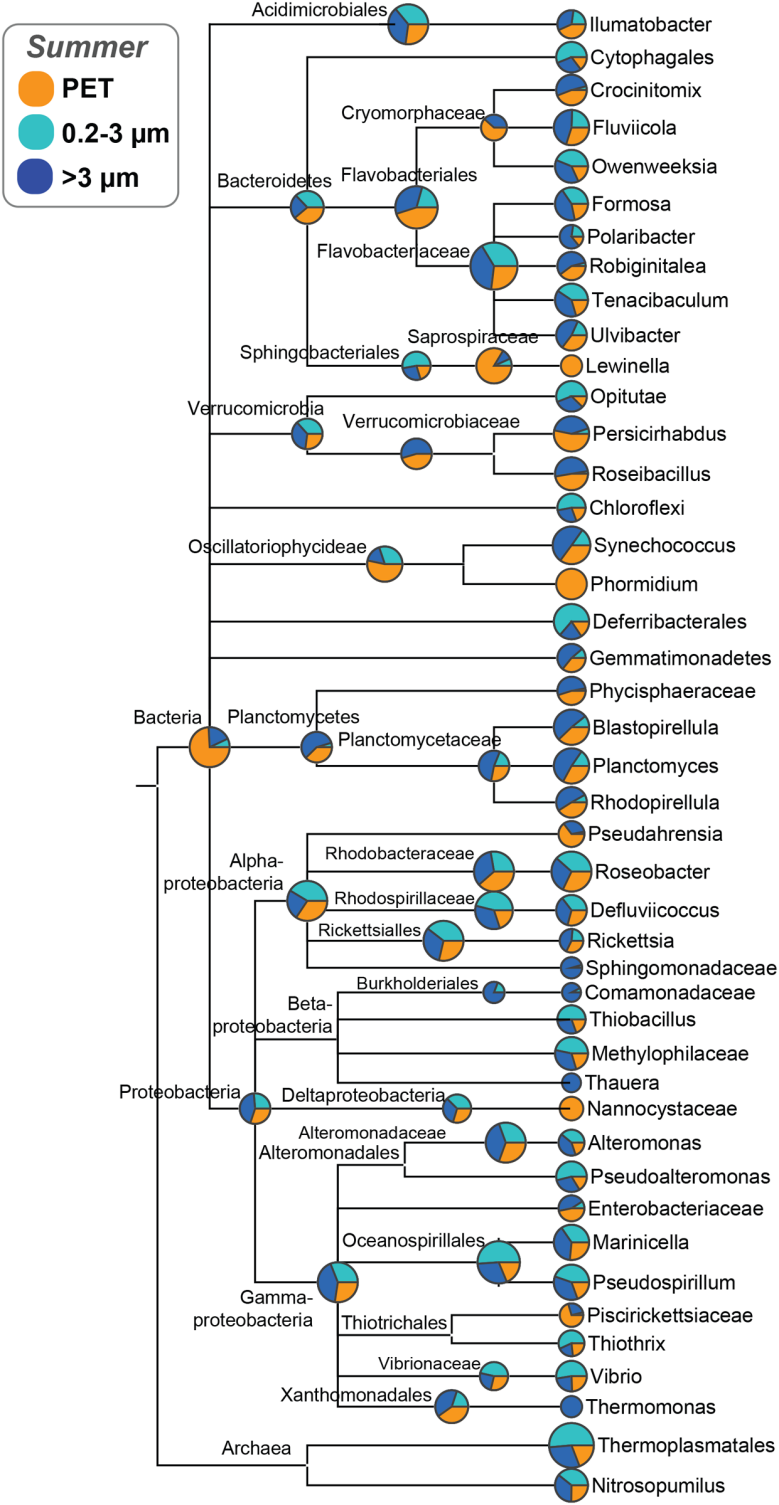
Phylogenetic representation and relative abundances of OTUs comprising PET-attached and seawater communities. Phylogenetic representation (based on 16S rRNA gene-based taxonomy assignment) of abundant OTUs (>0.5% of at least one community) and their relative abundances (pie charts based on log-scaled OTU counts) across treatments in summer.

The PET communities contained several hundreds of different operational taxonomic units (OTUs) per sample. OTUs assigned to the families Flavobacteriaceae, Cryomorphaceae, Saprospiraceae, and Rhodobacteraceae (Figs 3 and 4) were highly abundant within the PET-attached communities across all stations and seasons. Members of the genus *Tenacibaculum* (*Bacteriodetes*, *Flavobacteriaceae*) were the most dominant members of all of the PET communities. The genera *Crocinitomix* and *Owenweeksia* (both Bacteriodetes, Cryomorphaceae) were strongly represented on PET (S1 and S2 Figs), as well.

Multiple OTUs and taxonomic groups significantly discriminated the plastic biofilm communities from the seawater communities. The orders Sphingobacterales (Bacteriodetes) and Myxococcales (Deltaproteobacteria) significantly discriminated the PET-attached communities from either fraction of seawater across all stations (S4a Table), while an unclassified OTU from the Deltaproteobacteria was discriminant of the particle-associated seawater fraction. The free-living water community was discriminated by OTUs assigned to the Thermoplasmatales (Euryarchaeota), to the Roseobacter clade (Alphaproteobacteria, Roseobacteraceae), to the genus *Pseudospirillum* (Gammaproteobacteria, Oceanospirillaceae) and to the Thiotrichales (Gammaproteobacteria; S4 Table).

**Fig 5:**
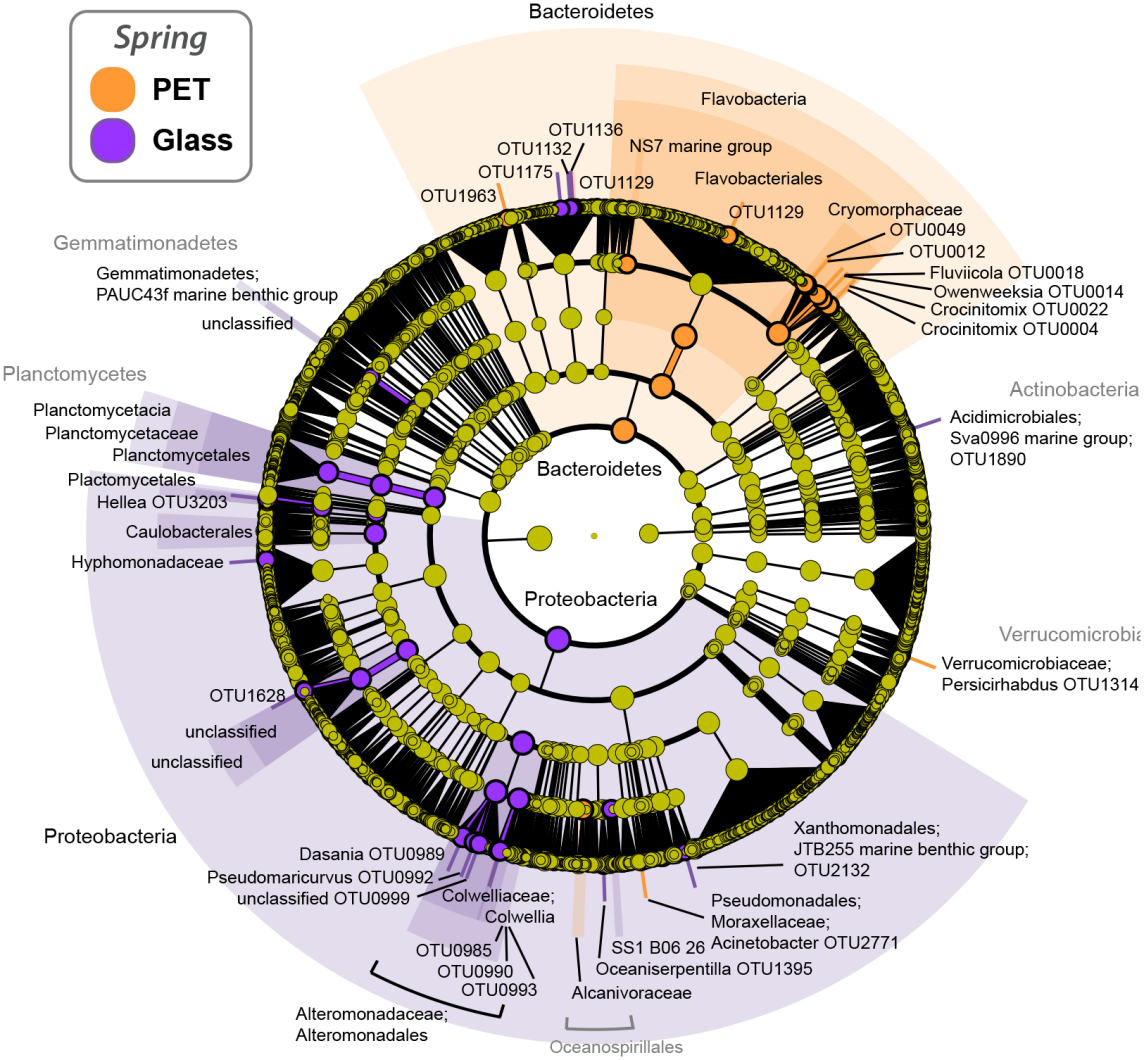
PET and glass biomarkers identified by linear discriminant analysis (LDA, LEfSe). Representation of taxa significantly discriminant of either PET- or glass-attached communities across all stations after 5–6 weeks incubation in the North Sea. See S4 Table for complete list and statistical summaries.

Despite the lack of significant difference between PET and glass total communities, differences were observed at the OTU level. OTUs assigned to the genera *Crocinitomix*, *Owenweeksia* and *Fluviicola* (all Bacteriodetes, Cryomorphaceae), *Acinetobacte*r (Gammaproteobacteria, Moraxellaceae) and *Persicirhabdus* (Verrucomicrobia, Verrucomicrobiaceae) were significantly discriminative of the PET communities (as opposed to glass) at all stations (Fig 5, S4b Table). At the family level, members of the Cryomorphaceae (Bacteriodetes) and Alcanivoraceae (Gammaproteobacteria) were significantly discriminative of PET communities. *Hellea* (Alphaproteobacteria, Hyphomonadaceae), *Dasania* (Gammaproteobacteria, Altermonadaceaea) and *Colwellia* (Gammaroteobacteria, Colwelliaceae) were significantly discriminative of glass communities (Fig 5, S4b Table).

**Fig 6:**
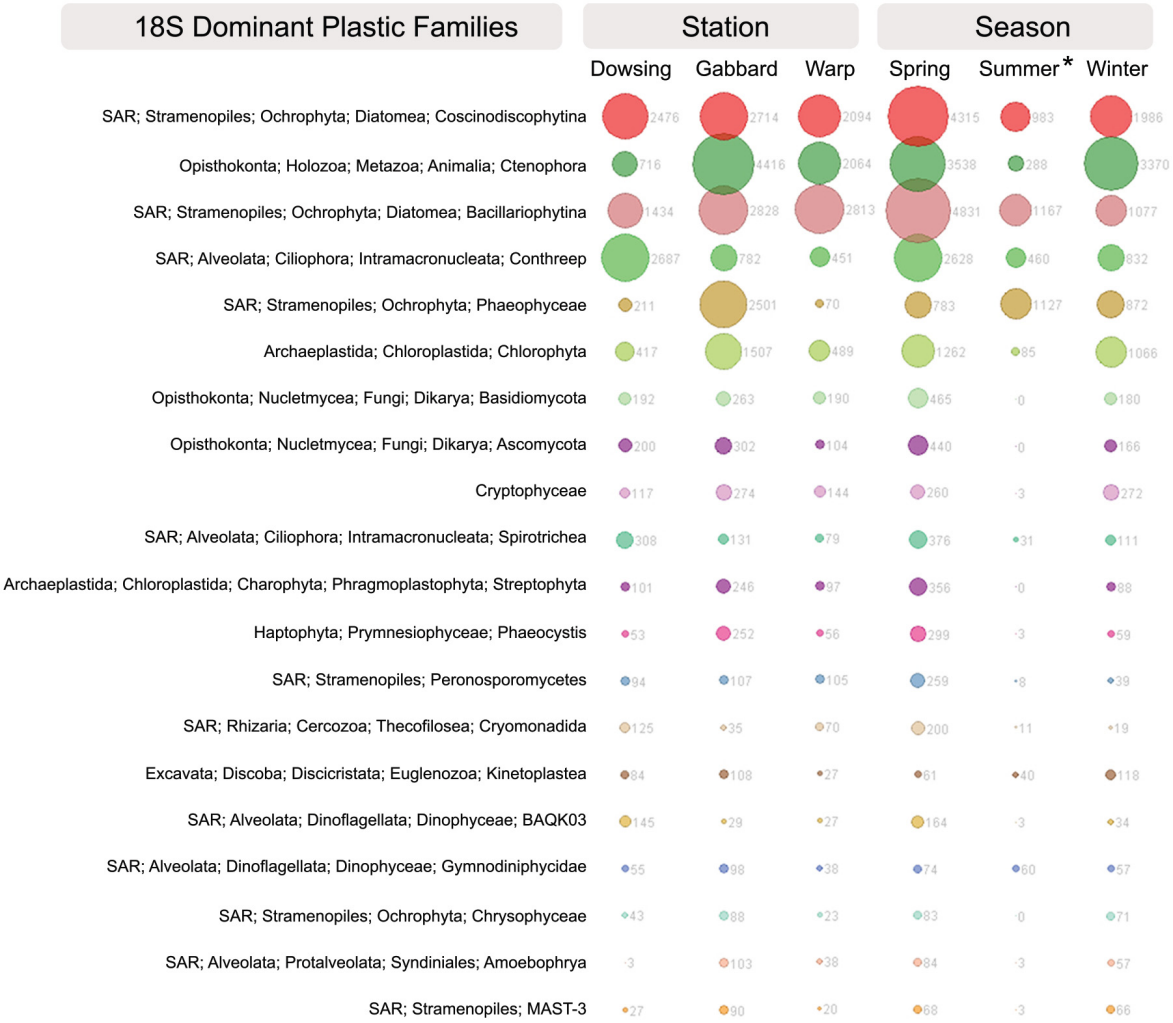
Abundant eukaryotic families within PET communities. Most abundant (top 25) eukaryotic families present in PET-attached biofilm communities after deployment in the North Sea for 6 weeks, grouped per deployment site/station and season (based on 18S rRNA gene analysis).

Eukaryotes were readily visible living attached to the PET surface (S3 Fig). The most abundant OTUs within the eukaryotic PET-attached community were assigned to the diatom groups, Coscinodiscophytina and Bacillariophytina, the brown algae Phaeophyceae, the ciliate group Conthreep and the green algae Chlorophyta (Fig 6). Many OTUs were classified as general metazoa (Fig 6).

Fungal taxa were noticeably more prevalent in the hard surface-colonizing (plastic and glass) biofilms than in either fraction of seawater (S4 Fig). These fungal colonizers are represented by 14 OTUs of unclassified Ascomycota, 11 OTUs of unclassified Basidiomycota and 2 OTUs of unclassified Chytridiomycota (S4 Fig). 19 of these OTUs were exclusively substrate-associated, with only 8 found in seawater samples (S4 Fig).

Discriminant of the PET communities as compared to seawater communities was an OTU belonging to the Bacillariophytina (super-group Stramenopiles-Alveolates-Rhizaria, SAR). The ciliate class Spirotrichea (SAR) was discriminant of the free-living seawater community. Several OTUs belonging to the Dinoflagellata (SAR) and *Phaeocystis* (Haptophyta) were discriminant of the particle-associated seawater community (S5a Table).

Although the overall variation between glass- and PET-attached eukaryotic communities was not significant (p = 0.29, Table 1), several OTUs were uniquely discriminant of either glass or plastic surfaces (S4 Table). Two unclassified OTUs were significantly discriminant of PET, while on glass three unclassified OTUs and OTUs belonging to Bacillariophytina, Dinophyceae and the group Dinoflagellata (all SAR) were significantly discriminant.

### Relationship between bacterial/archaeal and eukaryotic plastic assemblages and their environment

A Mantel test between the 16S and 18S OTU dissimilarity matrices indicate that the composition of the bacterial/archaeal and eukaryotic community assemblages were significantly positively correlated (r-statistic 0.901; p = 0.0001). A Mantel test between the Euclidean distance matrices of water properties (T, S, F; S1 Table) and the Bray-Curtis distance matrices from the OTU tables indicated that the bacterial/archaeal community structure was significantly positively correlated with temperature (r-statistic 0.5637; p = 0.012) and salinity (r-statistic 0.518, p = 0.034), but not with chlorophyll fluorescence (r-statistic -0.2204; p = 0.825). The eukaryotic community structure was significantly positively correlated with salinity only (r-statistic 0.6111; p-value, 0.013).

### Spatial and seasonal variation in PET biofilm communities

The community structure of PET-colonizing biofilms was influenced by both spatial and seasonal factors. PET-colonizing bacterial/archaeal (16S) communities were significantly different between stations and seasons. Between-station comparisons indicated that PET communities sampled at the geographically distant Dowsing were significantly distinct from those at both Warp and Gabbard (p = 0.001 and 0.002, respectively). The difference between these neighboring stations, Warp and Gabbard, was not significant (p = 0.095). Significant differences were found between all seasons (all p = 0.001). These relationships were visualized in the sample clustering patterns in principle coordinate (PCO) ordination (S5 Fig). PERMDISP analyses (main and pairwise, Table 1, S3 Table) of the 16S rRNA gene data for season, station, and treatment indicated no significant dispersion (all p > 0.05), except for the main test (i.e., not pairwise) of station in summer (p = 0.005) and the pairwise comparison Dowsing-Warp in summer (p = 0.002). In a comparison of OTUs across the PET-attached communities, several OTUs were significantly discriminant (p<0.05) of individual sample sites or seasons (S4 Table). For instance, the Vibrionaceae family was significantly represented at Dowsing across all seasons (S4d Table). In spring, OTUs assigned to the genera *Tenacibaculum* and the family Cryomorphaceae (both Bacteriodetes, Flavobacteriales) were highly significantly discriminant, while also discriminant were *Oleispira* (Gammaproteobacteria, Oceanospirillaceae), *Owenweeksia* (Bacteriodetes, Cryomorphaceae), *Ulvibacter* (Bacteriodetes, Flavobacteriaceae), and the family Alcanivoracaceae (Gammaproteobacteria; S4c Table). In winter, *Ornithinimicrobium* (Actinobacteria, Intrasporangiaceae) was significantly discriminant, with *Psychrobacter* (Proteobacteria, Moraxellaceae) and *Maribacter* (Bacteriodetes, Flavobacteriaceae) also discriminant (S4 Table).

Eukaryotic community assemblages followed similar patterns as was seen for bacterial/archaeal communities. The PET-attached communities varied significantly with season (p = 0.002; Table 1) and station (main PERMANOVA, p = 0.001). All communities differ significantly between seasons (winter-spring p = 0.028, winter-summer p = 0.03, spring-summer p = 0.045, S3 Table). Further, the communities sampled at Dowsing significantly differed from the communities at Warp and Gabbard (Warp-Dowsing p = 0.011, Gabbard-Dowsing p = 0.001, Warp-Gabbard p = 0.239, S3 Table). All 18S-based PERMDISP calculations (main and pairwise) of season, station, and treatment indicated that data were not significantly dispersed within groups (all p>0.05). Comparing the PET communities between seasons, the genus *Phaocystis* (Haptophyta) was significantly discriminant of spring samples, while in summer the super-group SAR was significantly discriminant (S4 Table).

## Discussion

### Marine biofilm communities on plastic resemble those on other surfaces

Plastic-attached communities were different from ‘free-living’ seawater communities, but not distinct from particle-associated or glass-attached communities. This is the first time this observation has been reported, as previous studies have restricted their analyses to comparisons between marine plastic biofilms and free-living seawater communities only [26,27,36]. It is not surprising that free-living and “attached” communities (either plastic-attached or >3 μm) differed significantly (Table 1), as the free-living/particle-associated dichotomy has been recognized for some time [63] across various marine biomes [64–67] and is thought to represent contrasting marine microbial lifestyles [68]. Further, the lack of significant difference between particle-associated and plastic biofilms (Table 1, Fig 2) suggests the driver of biofilm community composition is availability of a surface to colonize, rather than the composition of the surface.

In contrast with our second hypothesis, the PET-colonizing biofilms were not significantly distinct from those found on glass biofilms. Previous studies support these findings, in that no significant differences were found between communities that formed on “hard” substrates in freshwater [36] and marine [69] incubations, where ceramic, glass, plastic, aluminum, and coral skeleton were tested. Significant differences in community composition were found between soft (e.g., leaf litter, cardboard) and hard (e.g., tile, glass, plastic, aluminum) substrates [36]. This may in part be due to differences in their recalcitrance as microbial communities able to degrade soft but not hard substrates are selected for. The similarities between the PET and particle-associated (Fig 2; Table 1) and glass (Table 1) communities suggest that the plastic-colonizing communities are comprised of non-specific surface-colonizing microbes. This implies that the availability of PET as substrate did not play a major role in structuring the plastic-associated biofilm communities.

### Plastic-specific biofilm community features

Despite the similarities between PET and particle associated communities, a few notable OTUs uniquely discriminated PET from the other “attached” (3 μm and glass) communities across all stations (S4 and S5 Tables), indicating that factors in addition to surrounding water (e.g., station location and season) shape PET communities. Among OTUs that discriminated plastic biofilms from both seawater fractions across all stations were members of the Sphingobacteriales (Saprospiraceae, in particular) and Myxococcales (S4 Table), both of which were also described on North Atlantic plastic biofilms [26]. Members of the Sphingobacteriales have also been reported on other marine (e.g., coral [70], kelp [71]) and microbial surfaces; Saprospiraceae colonize filamentous microorganisms in activated sludge [72]. Their ability to produce exopolysaccharides [73] and scavenge biofilm materials for energy and carbon [72,74] likely make Sphingobacteriales successful biofilm community members. Myxobacteria excrete a polymeric substance to enable their gliding and swarming, as well as complex bioactive secondary metabolites and hydrolytic enzymes to lyse and degrade other bacteria and eukaryotes, which may help them compete for limiting resources in a biofilm environment [75]. Although Myxobacteria are typically associated with soil environments, a unique marine clade was first described in North Sea sediments [76].

Two genera and one family were present on PET (>0.5%; Fig 4) and absent from all seawater in summer, *Phormidium* (Cyanobacteria, Oscillatoriophycideae), *Lewinella* (Sphingobacteriales, Saprospriaceae), and Nannocystaceae (Myxococcales, features described above). Notably, all were present also on North Atlantic plastics sampled in the late spring and summer [26]. *Phormidium* are filamentous cyanobacterium that are most commonly found in benthic cyanobacterial mats and are known to degrade hydrocarbons [77]. In contrast with their ubiquity as plastic biofilm members, *Phormidium* were absent from the seawater of the North Atlantic study [26]. *Lewinella* has been found as a defining member of biofilms colonizing red macroalgae [78]. Also discriminant of the PET communities relative to seawater was the diatom group, as opposed to dinoflagellates, which served as biomarkers for the particle-associated seawater community (S5 Table). Diatoms are known to be among the first recruits in marine biofilm formation and likely prime surface conditions for subsequent heterotrophic microbial colonizers [79–81].

Members of the Verrucomicrobia phylum were identified in both the attached (plastic and >3 μm) and free-living microbial communities and their distribution indicated that ecologically distinct lineages within this diverse phylum occupy unique niches in the marine environment. In this study, the Verrucomicrobiae family (Verrucomicrobia subdivision 1) was significantly discriminant of PET-attached communities and was also abundant on plastic collected from the Northern Atlantic [26]. Contrastingly, the Oppitutae family (Verrucomicrobia subdivisions 3 and 4) was significantly discriminant of free-living communities (S4e Table). This evidence supports one of the first and only studies to add ecological context to the diverse lineages within the Verrucomicrobia [82]. This work identified subdivision 4 as a free-living lineage that dominates ocean surface waters and contrasts with subdivision 1, which dominates sediment samples. Our study supports this dichotomy and extends the current view to suggest subdivision 1 to specialize in particle-attached lifestyles.

### Potential bacterial specialization in complex carbon degradation

Bacterial families that comprised >0.5% of the communities on our deployed North Sea plastics and that were described previously on North Atlantic plastic fragments [26] include Saprospiraceae, Flavobacteriaceae, Rhodobacteraceae, Alteromonadaceae, Oscillatoriaceae (Figs 3 and 4, S1 Fig). All of these families include members known to degrade complex carbon substrates and specialize in marine surface-associated or biofilm/microbial mat lifestyles [77,82–86]. Newly found here is the importance of families such as Cryomorphaceae (namely, genera *Crocinitomix*, *Owenweeksia*, and *Fluvicola*), Planctomycetaceae, and Verrucomicrobiaceae (namely, genus *Persicirhabdus*) in the plastic-colonizing communities (Figs 3 and 4, S1 Fig). Members of the mostly marine Cryomorphaceae family have been found to contain dioxigenases and haloacid dehalogenases that may indicate a role of these microbes in the respiratory degradation of recalcitrant compounds [87]. Further, members of the Cryomorphaceae tend to be catalase-positive and carotenoid producing, traits that can protect from oxidative stress (e.g., thermal, UV [87]). This trait may be beneficial for microbial communities attached to floating plastic and may contribute to this group being the second most abundant on PET across all seasons and stations (Fig 3).

Notably, members of the Rhodobacteraceae and Alteromonadaceae are known hydrocarbon degraders [88,89] and a member of Rhodobacteraceae (*Rhodococcus ruber*; [90]) has been reported to degrade polyethylene in biofilms [90]. Though PET is a more stable substrate than polyethylene, these parallels offer tempting speculation about the ability of plastic biofilm communities to degrade the polymers they colonize. However, these microbial groups are very diverse and we are far from understanding the metabolic potential of these biofilm communities to the extent that would allow us to model potential plastic-degrading pathways. Interestingly, the family *Alcanivoraceae* was discriminant of the PET-communities across all stations in spring and is among those that discriminate PET from glass communities. This family’s type genus is the biofilm-forming hydrocarbonoclastic *Alcanivorax*, which has been identified in marine waters worldwide and has the genomic capacity to degrade a range of oil-derived hydrocarbons [91]. Further studies are needed to interrogate the metabolic potential of plastic-dwelling microbes to degrade plastics and plastic-bound organic pollutants.

### Plastics as a refuge for potential pathogens

Roughly one third of the plastic-associated bacterial or archaeal sequences identified in this study were assigned to the genus *Tenacibaculum* (Flavobacteriaceae), which is a diverse genus with at least 20 species described. Our own study defined 78 *Tenacibaculum* OTUs (97% intra-OTU similarity), capturing notable microheterogeniety even in the short V4 region of the 16S rRNA gene. This heterogeneity may explain why the genus was found in the seawater, though most known *Tenacibaculum* show a preference for an attached lifestyle [92]. Further, the genus *Tenacibaculum* harbors several fish pathogens [92] and warrants consideration of the potential for plastics to serve as vectors for pathogenic microorganisms. Fish that consume plastic may ingest the harmful colonizers [93]. The potential for plastics to serve as vectors for possible pathogenic microbes has been documented previously [26,27]. *Vibrio*, a genus also present in the plastic-associated communities from this study, was identified on North Atlantic plastics [26]. Potential pathogens were identified on microplastics collected in an urban river, especially members of Campylobacteraceae, which were highly abundant on plastics collected near a wastewater treatment plant [27]. Pathogenic microbes tend to specialize in opportunistic and surface-associated lifestyles to optimally exploit their hosts and have been described on aquatic aggregates previously [94,95]. Plastic particles may be particularly well suited as a refuge for their recruitment, attachment, and subsistence. It is likely that potentially pathogenic microorganisms will continue to be identified as members of biofilms on aquatic debris and aggregates. However, the short V4 region of the 16S rRNA gene that this and past studies are based on does not provide enough taxonomic information about the serotypes nor their function to infer pathogenicity, thus supplemental approaches are necessary.

### Algal and fungal components of plastic biofilms

Diatoms have been found attached to surfaces of ocean plastics [30,34], where they can be the most abundant type of eukaryote [96]. Diatoms have specific assemblages of co-associated bacterial epibionts [97] with a handful of genera repeatedly reported across numerous studies [98]. Among those, *Roseobacter*, *Alteromonas*, and *Pseudoalteromonas* are also dominant in the plastic communities here. Further, Flavobacteria, namely *Tenacibaculum* and *Polaribacter*, two genera found in the PET biofilms here, are major colonizers of diatom detritus [99]. This interdependence likely contributed to the strong correlation between the 16S and 18S rRNA gene dissimilarity matrices.

Fungi represent an unexplored component of the aquatic plastic microbiome. We found a high prevalence of fungal OTUs on plastic and glass relative to either fraction of seawater, though the minimal information in the short 18S rRNA V9 region used in this study offers little taxonomic resolution and insight into the fungal populations (S4 Fig). Nonetheless, this trend implicates hard substrates as ideal habitats for fungal growth. As more than half of the total fungal OTUs appear to be hard-substrate-specific, these surfaces may be potential hotspots for fungal diversity and functional novelty in ocean systems. Endophytic fungi (Ascomycota; *Pestalotiopsis*) isolated from the Ecuadorian Amazonian rainforest were found to efficiently degrade and use as their sole carbon source polyester polyurethane under both aerobic and anaerobic conditions [100]. The role and activity of fungi in ocean plastic biofilms are prime candidates for future study.

### Spatial and seasonal influences on the plastic microbiome

Consistent with our hypotheses, biofilms formed on plastics from different sites had significantly different community structures. However, the microbial community similarity followed a trend in the physical proximity of the stations. The biofilm and seawater communities at the proximal Warp and Gabbard stations were not significantly different from each other, yet they both differed significantly from those at the more distant Dowsing station (Table 1; Fig 2). This pattern may reflect the influence of local physico-chemical conditions (S1 Table), which are likely to be largely driven by contrasting water masses (e.g., Scottish coastal versus Channel water) in the North Sea (Fig 1). Numerous studies have found community structure to differ more between sites than between different substrates [36, 79, 101], supporting our conclusion that location is the greatest driver of community composition. Recently, the first multi-ocean basin biogeographical survey of the plastic microbiome supported this experiment-based trend: microbial communities differed more between ocean basins than between plastic types [102].

To specifically determine the extent to which the seasonal and spatial factors uniquely influence the PET biofilm communities relative to the background seawater and glass-associated communities, one would need to compare these three communities across all seasons and at all study sites. Due to technical challenges that impeded our study design (Fig 1), this comparison was not possible, but could be considered for future studies.

Taken together, the seasonal and spatial patterns suggest that the plastic surface environment at the polymer-water interface does not exert strong enough selection to drive species sorting to overcome other niche-defining factors. If the plastic selects for some unique microbial constituents (e.g., polymer-consuming microbes), the approach used here to study the overall community may not provide sufficient resolution, as plastic-influenced organisms may be minor community members. As biofilms matured over a 6-week incubation, only the initial recruits would have direct contact with the polymer surface; later recruits are more likely to interact with existing biofilm members and the abiotic components of the biofilm matrix or surrounding seawater. Such a scenario would result in a generalized marine particle/surface-associated community when the community members are studied en masse, as was done and found here.

## Outlook

Understanding plastic-dwelling biofilms at increased spatial resolution will improve our knowledge of biofilm community assembly processes, as well as plastic-microbe interactions, such as the potential for members of the plastic microbiome to degrade plastic hydrocarbons. Future studies should include other major plastic polymers in addition to PET, e.g., polyethylene, polypropylene, polystyrene or polyamide and may benefit from longer incubation periods (6–12 months minimum) to allow for development of possible degrading populations. Three-dimensional confocal fluorescence *in situ* hybridization microscopy of labeled community members could help discern which plastic biomarkers are specifically attached to the plastic surface, versus those that are attached to other biofilm members (e.g., diatoms) or abiotic components (e.g., exopolysaccharides). Also, careful processing of the plastic samples to discretely remove the bulk biofilm and leave the specifically attached or embedded microbes could be attempted. For lab-based confirmation of degradation, enrichment cultures from the marine communities should be developed to track degradation products, as has been successfully applied in other plastic degradation studies [90,103,104,105]. A bacterium was recently shown to both degrade and assimilate PET, *Ideonella sakaiensis* [106]; notably, this genus was not found in our study. Biofilms are target communities to probe for future discovery of novel plastic-degrading microbes and the genes involved in the enzymatic process. Biofilms enable microbes to utilize non-soluble substrates and most, if not all, isolated microbial plastic degraders have been biofilm formers (isolated degraders summarized in SI of [104]).

The rate of plastic debris accumulation in aquatic ecosystems is increasing and will persist for long time scales [12]. While identifying the major sources and sinks of plastics are a critical and on-going topic of study [12], the plastic microbiome is a novel and near-permanent feature of global oceans that also warrants greater investigation.

## Supporting Information

S1 Fig Genus-level representation of OTUs identified in PET-attached biofilms. Identified bacterial/archaeal genera (16S rRNA gene) comprising PET-attached biofilms across all stations and seasons sampled. OTU counts have been square root transformed.
(PDF)

S2 Fig Phylogenetic representation and relative abundances of OTUs comprising PET-and glass-attached communities. Phylogenetic representation (based on 16S rRNA gene-based taxonomy assignment) of abundant OTUs (>0.5% of at least one community) and their relative abundances (pie charts based on log-scaled OTU counts) attached onto PET and glass substrates in spring.
(PDF)

S3 Fig Scanning electron micrograph of marine biofilm members on plastic bottles. Eukaryotic biofilm members living at the surface of a PET plastic bottle after incubation for 5–6 weeks in the coastal North Sea. (a) Diatom members of PET-colonizing community. (b) A mass of interacting eukaryotes (diatoms, algae, possible ciliates) within the PET-colonizing biofilm community.
(PDF)

S4 Fig Abundance of Fungal OTUs across treatments. Bar graph representing the abundance of reads assigned to fungal OTUs across all treatments (PET-attached, glass-attached, 0.2–3 μm seawater, >3 μm seawater). OTU counts are normalized to the number of samples of each treatment to account for unbalanced representation of each sample type.
(PDF)

S5 Fig Principle Coordinate Ordinations relating variation in plastic (PET) microbiome community composition across season and station variables. PCOs representing similarity of biofilm communities based on counts of OTUs across samples (16S/18S rRNA gene data, see methods for OTU definition). Displayed are comparisons of (a) bacterial/archaeal and (b) eukaryotic PET-attached communities sampled across winter, spring, and summer and Dowsing, Warp, and Gabbard stations.
(PDF)

S1 Table Environmental data (temperature, salinity, chlorophyll fluorescence) and season-sample pairs. Average temperature, salinity, and chlorophyll fluorescence data for each season-station pair used in the dissimilarity matrix for Mantel test. Data were continuously collected by the SmartBuoy system at each station the substrates were deployed and the water was collected. Dowsing-Summer dropped from Mantel analysis, as no data were available for salinity.
(XLSX)

S2 Table Primer constructs used for amplicon sequencing, including indices (i5/i7) used. See Kozich et al, 2013 for dual indexing strategy.
(XLSX)

S3 Table PERMANOVA/PERMDISP results of pairwise tests comparing bacterial/archaeal (16S rRNA gene) and eukaryotic (18S rRNA gene) community structure across different seasons, stations, and treatments. PERMANOVA was performed on Bray-Curtis dissimilarity matrices based on OTU counts across microbial communities (a: bacterial/archaeal, 16S rRNA gene; b: eukaryotic, 18S rRNA gene). Significant results (P < 0.05) highlighted in bold and noted by **. Factors include station (Warp, Gabbard, Dowsing), treatment (PET, >3 μm, 3–0.2 μm seawater fractions in summer) and season (spring, winter, summer). P-values were obtained using type III sums of squares and 999 permutations [‘p(perm)’] or calculating Monte-Carlo tests [‘p(MC)’]. Unique perm, unique permutations. The results (p values) of the PERMDISP tests calculated to centroids are also provided.
(XLSX)

S4 Table Results of linear discriminant analysis test to identify differentially abundant bacterial/archaeal OTUs for treatment, station, and season comparisons using LEfSe. Discriminant OTUs (based on16S rRNA gene analysis) identified, using class and subclass distinctions, in comparisons of: (a) PET-associated, particle-attached (>3 μm) and free-living (0.2 μm-3 μm) seawater communities in summer (lefse class: treatment) across all stations (lefse subclass: station), (b) PET- and glass-attached communities in spring (lefse class: treatment) across all stations (lefse subclass: station), (c) PET-associated communities from summer, spring, winter (lefse class: season) across all stations (lefse subclass: station), (d) PET-associated communities from Dowsing, Warp, Gabbard (lefse class: station) across all seasons (lefse subclass: season), (e) attached (PET and >3μm seawater fraction) and free-living (3–0.2 μm seawater fraction) communities (PET and 3.0 μm; lefse class: treatment) across all stations (lefse subclass: station).
(XLSX)

S5 Table Results of linear discriminant analysis test to identify differentially abundant eukaryotic OTUs for treatment, station, and season comparisons using LEfSe. Discriminant OTUs (based on18S rRNA gene analysis) identified, using class and subclass distinctions, in comparisons of: (a) PET-associated, particle-attached (>3 μm) and free-living (0.2 μm-3 μm) seawater communities in summer (lefse class: treatment) across all stations (lefse subclass: station), (b) PET- and glass-attached communities in spring (lefse class: treatment) across all stations (lefse subclass: station), (c) PET-associated communities from summer, spring, winter (lefse class: season) across all stations (lefse subclass: station), (d) PET-associated communities from Dowsing, Warp, Gabbard (lefse class: station) across all seasons (lefse subclass: season), (e) attached (PET and >3μm seawater fraction) and free-living (3–0.2 μm seawater fraction) communities (PET and 3.0 μm; lefse class: treatment) across all stations (lefse subclass: station).
(XLSX)
