# Supplementary figures and images for: Microbes on a Bottle: Substrate, Season and Geography Influence Community Composition of Microbes Colonizing Marine Plastic Debris
Source: PLoS One. 2016 Aug 3;11(8):e0159289. doi: 10.1371/journal.pone.0159289 (PMC4972250; doi:10.1371/journal.pone.0159289)

# Genus-level taxonomy profile of PET-colonizing biofilms (16S)

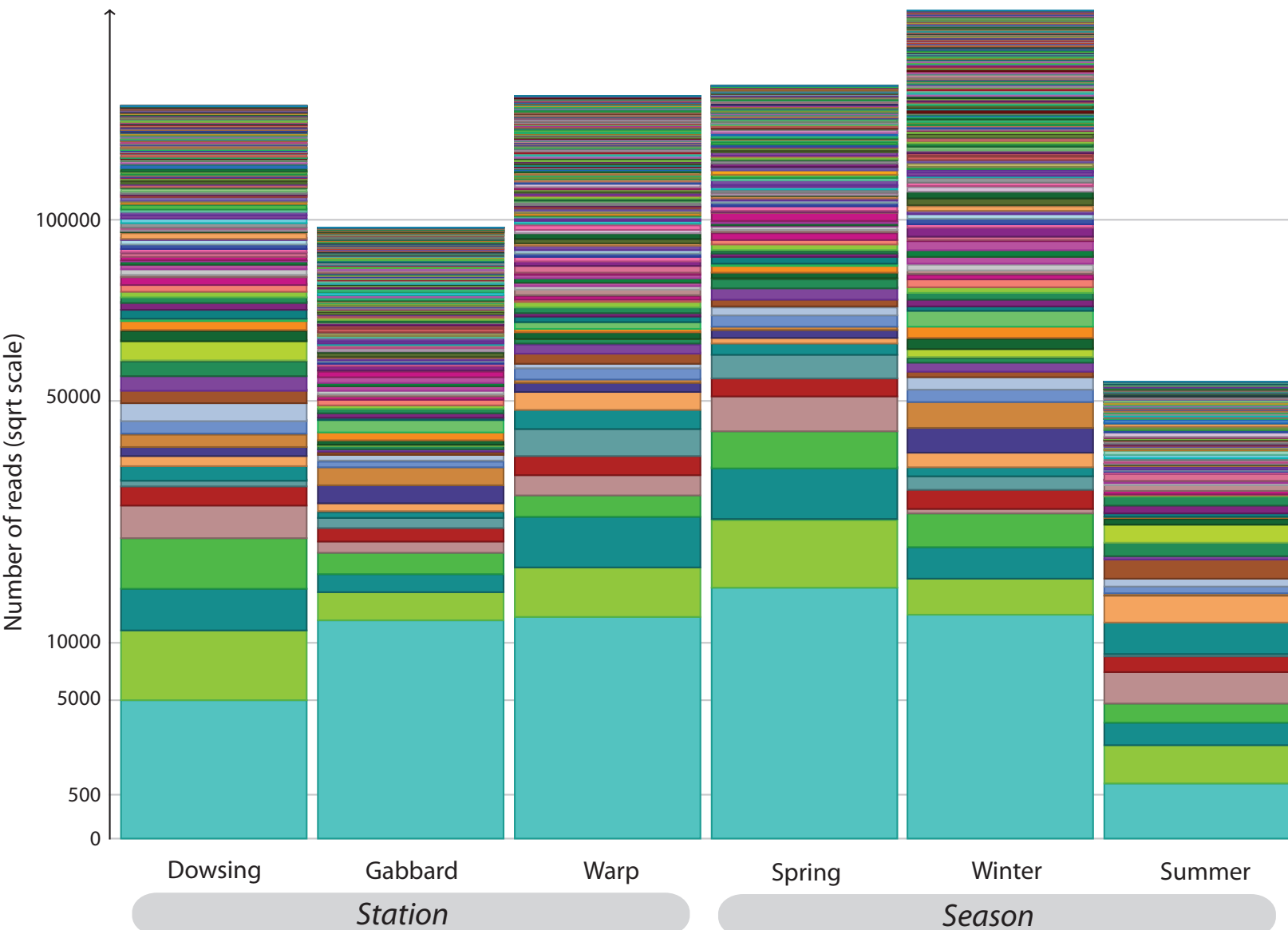

Legend (taxa in order of abundance):

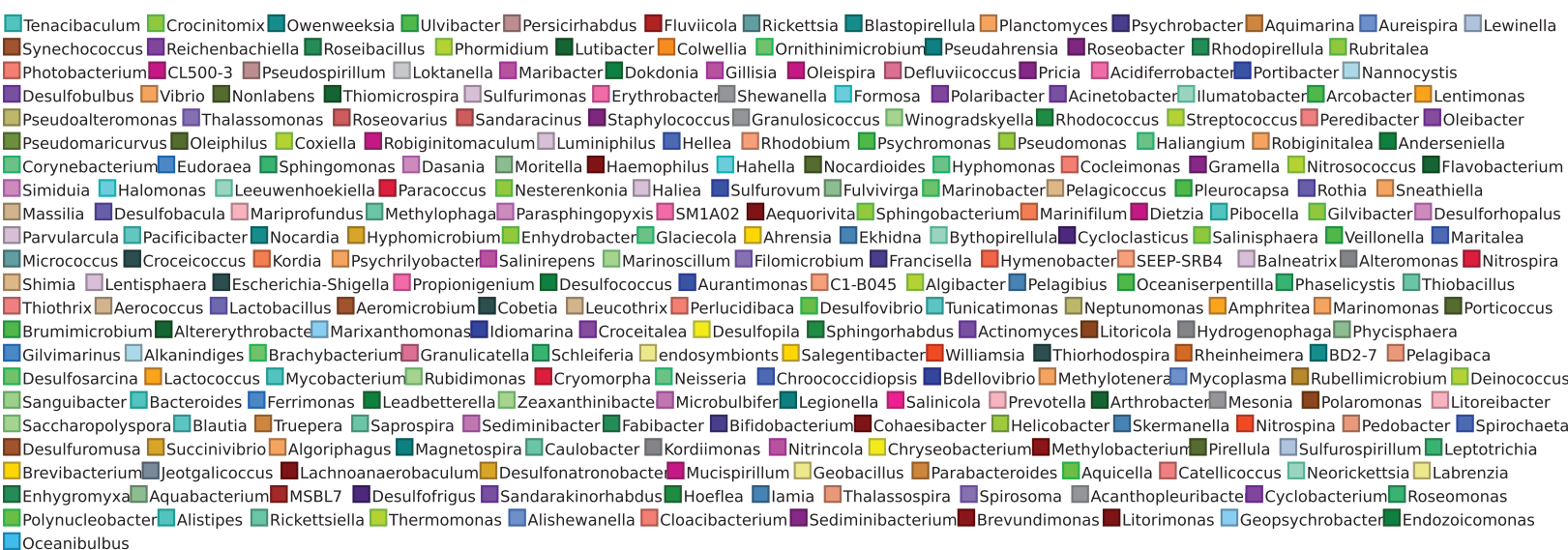

Supplement: S1 Fig — Identified bacterial/archaeal genera (16S rRNA gene) comprising PET-attached biofilms across all stations and seasons sampled. OTU counts have been square root transformed. (PDF) [file pone.0159289.s001.pdf]

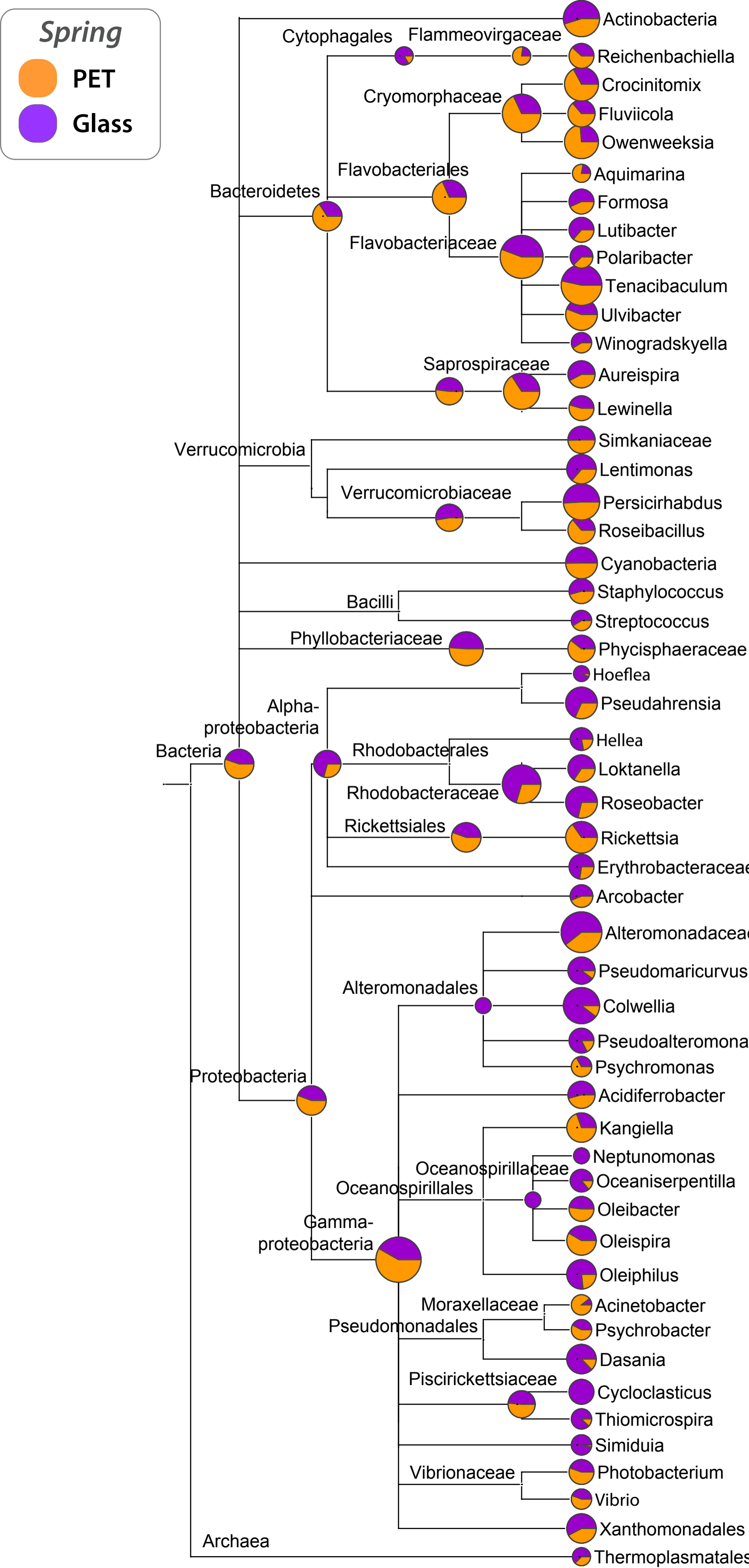

Supplement: S2 Fig — Phylogenetic representation (based on 16S rRNA gene-based taxonomy assignment) of abundant OTUs (>0.5% of at least one community) and their relative abundances (pie charts based on log-scaled OTU counts) attached onto PET and glass substrates in spring. (PDF) [file pone.0159289.s002.pdf]

(a)

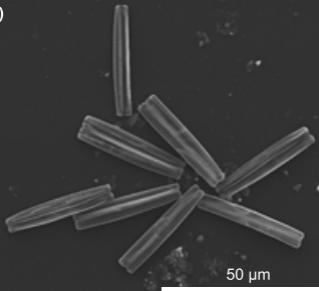

(b)

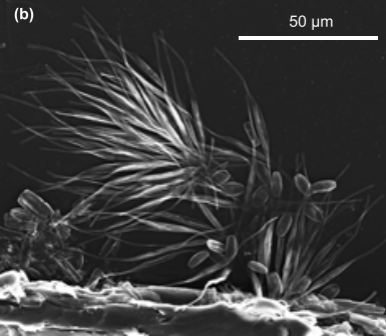

Supplement: S3 Fig — Eukaryotic biofilm members living at the surface of a PET plastic bottle after incubation for 5–6 weeks in the coastal North Sea. (a) Diatom members of PET-colonizing community. (b) A mass of interacting eukaryotes (diatoms, algae, possible ciliates) within the PET-colonizing biofilm community. (PDF) [file pone.0159289.s003.pdf]

# Fungal OTUs across all treatments

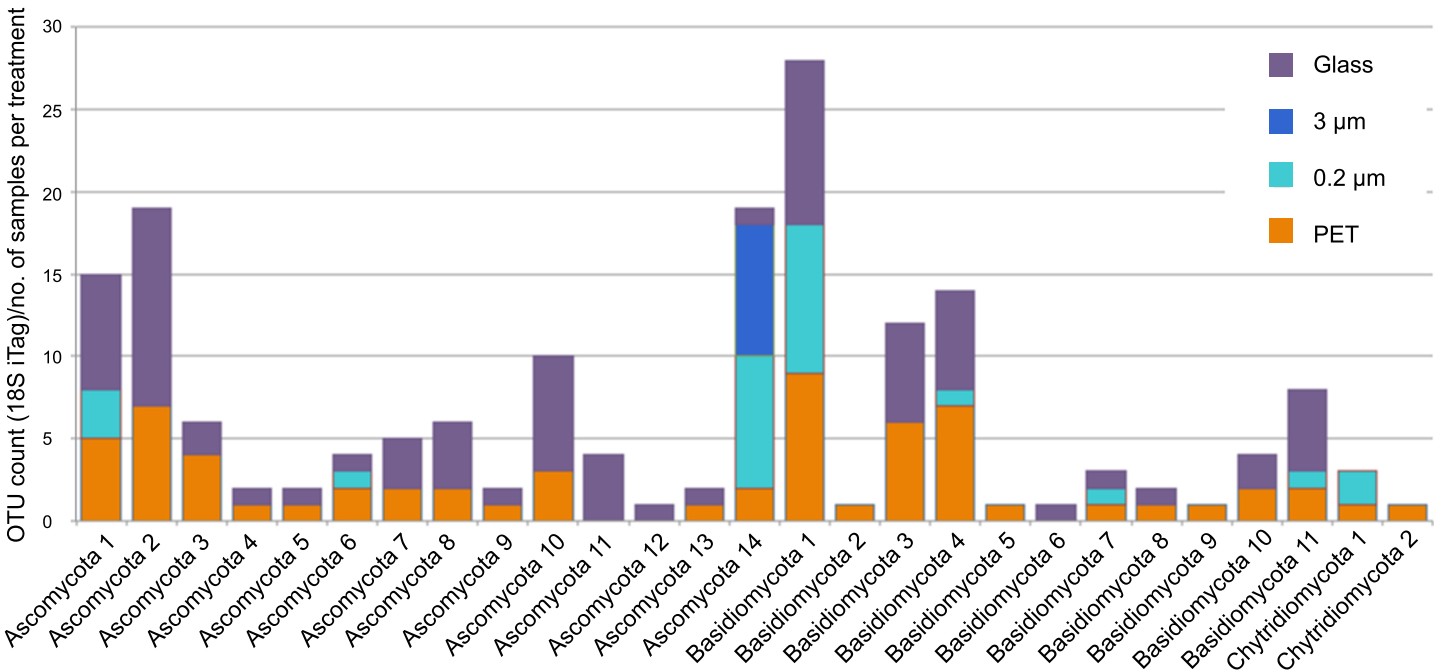

Supplement: S4 Fig — Bar graph representing the abundance of reads assigned to fungal OTUs across all treatments (PET-attached, glass-attached, 0.2–3 μm seawater, >3 μm seawater). OTU counts are normalized to the number of samples of each treatment to account for unbalanced representation of each sample type. (PDF) [file pone.0159289.s004.pdf]
