# Supplementary material for: Microbes on a Bottle: Substrate, Season and Geography Influence Community Composition of Microbes Colonizing Marine Plastic Debris
Source: PLoS One. 2016 Aug 3;11(8):e0159289. doi: 10.1371/journal.pone.0159289 (PMC4972250; doi:10.1371/journal.pone.0159289)
Supplement: S5 Fig — PCOs representing similarity of biofilm communities based on counts of OTUs across samples (16S/18S rRNA gene data, see methods for OTU definition). Displayed are comparisons of (a) bacterial/archaeal and (b) eukaryotic PET-attached communities sampled across winter, spring, and summer and Dowsing, Warp, and Gabbard stations. (PDF) [file pone.0159289.s005.pdf]

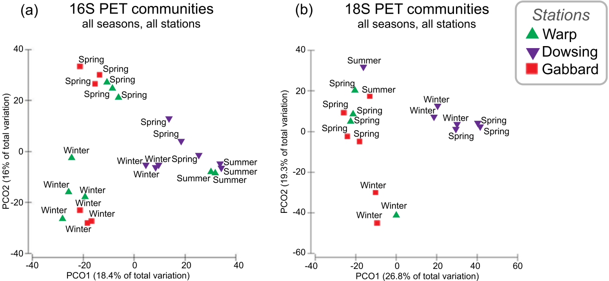

(a)
16S PET communities
all seasons, all stations
PCO1 (18.4% of total variation)
PCO2 (16% of total variation)
Spring
Summer
Winter
(b)
18S PET communities
all seasons, all stations
PCO1 (26.8% of total variation)
PCO2 (19.3% of total variation)
Spring
Summer
Winter
Stations
Warp
Dowsing
Gabbard
